# Early onset lactating adenoma and the role of breast MRI: a case report

**DOI:** 10.1186/1752-1947-3-43

**Published:** 2009-01-30

**Authors:** Stefano Magno, Daniela Terribile, Gianluca Franceschini, Cristina Fabbri, Federica Chiesa, Alba Di Leone, Melania Costantini, Paolo Belli, Riccardo Masetti

**Affiliations:** 1 Department of Surgery, Breast Unit, Catholic University, Policlinico "A. Gemelli", Largo Agostino Gemelli, Rome, Italy; 2 Department of Radiology, Catholic University, Policlinico "A. Gemelli", Largo Agostino Gemelli, Rome, Italy

## Abstract

**Introduction:**

Lactating adenoma is a benign condition, representing the most prevalent breast lesion in pregnant women and during puerperium; in this paper, a case of a woman with lactating adenoma occurring during the first trimester of pregnancy is reported. There have been no reports in the literature, according to our search, focusing on magnetic resonance imaging findings in cases of lactating adenomas. Also the early onset of the lesion during the first trimester of pregnancy is quite unusual and possibly unique.

**Case presentation:**

We report the case of a primiparous 30-year-old Caucasian woman, who noted an asymptomatic lump within her left breast during the 9^th ^week of gestation, slightly increasing in size over the next few weeks. Ultrasound demonstrated a hypoecoic solid mass, hypervascularized and measuring 4 cm. On magnetic resonance imaging, performed in the first month after delivery, the lesion appeared as an ovoidal homogeneous mass, with regular margins and a significant contrast enhancement indicative of a giant adenoma.

**Conclusion:**

Magnetic resonance imaging could play an important role in the differential diagnosis of pregnancy-related breast lumps, particularly during puerperium, thus avoiding unnecessary surgical biopsies.

## Introduction

Lactating adenomas are benign stromal alterations and represent the most prevalent breast lesions in pregnant women and during puerperium; nevertheless, any mass that appears during this period must be carefully evaluated to rule out a malignancy. Traditionally, ultrasound represents the main diagnostic tool of a breast lump during pregnancy because of its accuracy in the discrimination between solid and cystic lesions, and its safety due to the lack of radiation exposure. Cytologic and micro-histologic findings after percutaneous procedures often fail to exclude malignancy due to the lactational changes within the breast induced by the gestational hormonal milieu [1].

We report on a case of a woman with a lactating adenoma based on ultrasound (US) and magnetic resonance imaging (MRI) findings with clinical and pathologic correlation. In the literature, only in one case has previously reported of the ultrasound and magnetic resonance findings in a patient with a giant adenoma [2].

## Case presentation

Our patient, a primigravida 30-year-old Caucasian woman, noted an enlargement of her left breast, associated with an asymptomatic lump during the first trimester of her pregnancy but she only reported the finding to her doctor during the third trimester. She reported no history of breast cancer in her family and no personal history of breast pathology or any other remarkable medical facts. She had received no hormonal treatment in the past. Physical examination revealed a retroareolar lump, moderately deforming the skin profile in the upper quadrants of the left breast and soft in consistency. The mass was not fixed to the chest wall, but the overlying skin was slightly retracted. No regional adenopathy in the axillary and supraclavicular basins was detected.

**Figure 1:**
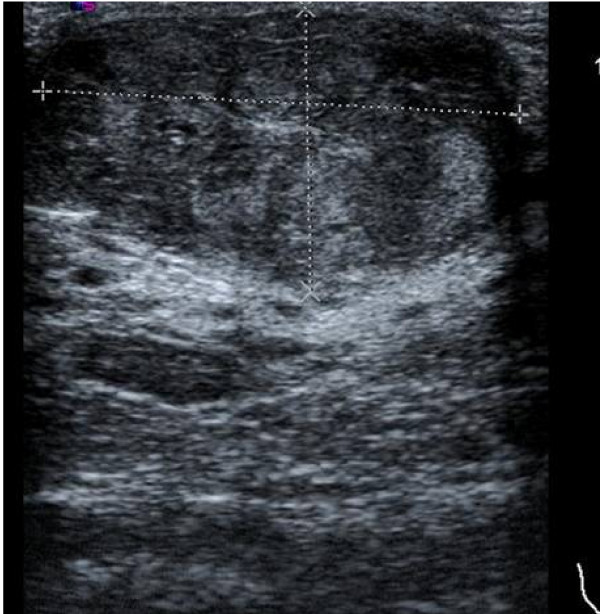
**Ultrasound evaluation of the lesion**.

A first ultrasound evaluation, performed during the last weeks of gestation, demonstrated a retroareolar hypoecoic heterogeneous solid mass, with regular margins, an oval configuration and measuring 3·7 cm. On Doppler sonography, a large number of vascular branches inside the lesion and a slight posterior acoustic enhancement were detected (Figure 1). Axillary lymph nodes appeared to be normal. After delivery, she began to breast-feed, but only from her right breast, since no milk could be elicited from the breast containing the mass. A repeat ultrasound on postpartum day three revealed a slight increase in the size of the mass (4 × 1·7 cm).

**Figure 2:**
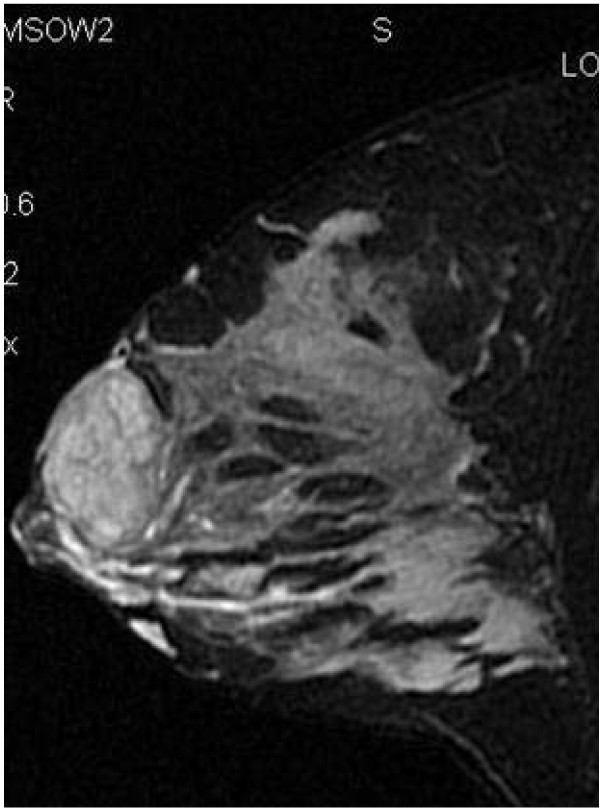
**MRI: STIR sequences with fat suppression in sagittal plane**.

Magnetic resonance imaging (MRI) was then performed, using a 1·5 T MRI system and a dedicated breast coil. The imaging was obtained before and after intravenous administration of gadolinium, using SPGR and STIR sequences, completed by MIP and MPR reconstructions. The high-resolution images after contrast medium showed dense breast tissue with diffuse enhancement, and a marked asymmetry between the two sides due to the asymmetrical lactation. The left breast showed much less blood flow and permeability compared to the contralateral breast, while the mass, containing ipointense septa, was seen displacing the normal breast parenchyma and the nipple-areolar complex inferiorly, compressing almost completely the galactiferous ducts, which appeared slightly widened in the inferior part of the gland (Figure 2).

The overall aspect of the lesion was considered benign, highly suggestive for giant fibroadenoma, but an open surgical biopsy was advised by the radiologist.

**Figure 3:**
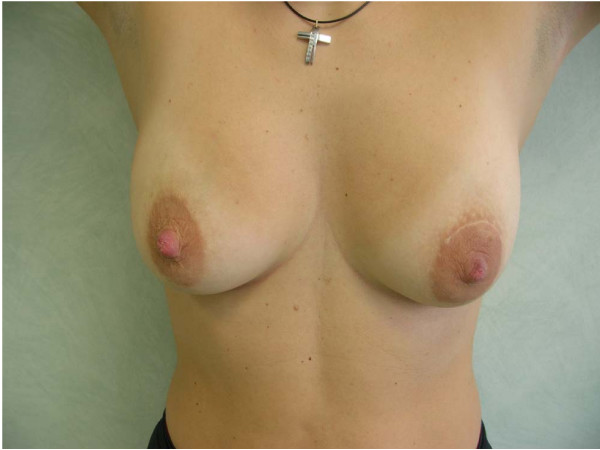
**Appearance of breast six months after surgery**.

A percutaneous fine needle biopsy was then proposed, but the patient refused; the intervention was planned approximately 2 months after delivery, under local anaesthesia. A periareolar superior approach was performed and the mass was removed trying not to interrupt the surrounding galactiferous ducts displaced and narrowed by the lesion. By remodelling the gland, the normal shape of the breast was restored (Figure 3).

At excisional biopsy, the mass appeared brown in colour, well circumscribed, with a lobulated surface, measuring 3·5 × 2·5 × 2·5 cm. Microscopically, a proliferation of benign ducts separated by sparse intervening stroma with preservation of lobular architecture was found.

Within 10 days of the surgery, a small amount (approximately 10 cc/die) of milky serum was drained through the surgical wound; 1 week later, the secretion stopped without any sequela on the scarring and subsequent healing of the breast and the patient continued to breast-feed for the next 6 months after surgery.

## Discussion

The aetiology of lactating adenomas remains unclear; the consensus at this time is that they are tubular adenomas with lactational changes. Necrosis and haemorrhage are not prominent features, occurring in only 5% of cases. Lactating adenomas are the most common masses occurring during pregnancy usually appearing during the third trimester of gestation and regress spontaneously after delivery. However, excisional biopsy is often required for the following reasons:

1) Unsatisfactory reliability of percutaneous bioptic procedures in the evaluation of pregnancy associated breast lesions makes diagnosis uncertain in most cases. In particular, fine needle aspiration may not have the same accuracy in pregnancy and puerperium due to the hyperproliferative state of the glandular tissue, thus increasing the risk for a false positive diagnosis [3].

2) The risk of an associated breast cancer is not negligible. Lactating adenomas are not thought to carry an increased risk of breast carcinoma, even though the lack of a large series regarding this issue keeps the question still unresolved. In any case, some cases have been reported of patients who developed both the lesions (lactating adenoma and invasive carcinoma) in the same site [4].

During pregnancy, high concentrations of oestrogen, progesterone and prolactin promote the growth of ducts and the formation of tubuloalveolar structures; progesterone and prolactin are known for their synergistic proliferative activity, playing a defined role in murine and human breast cancer. Lactating adenomas have been shown to express high amounts of prolactin receptors, whose stimulation in a fully primed breast, as a result of lactation, could promote rapid growth of existing foci of breast cancer cells. Close follow-up should be maintained in women with lactating adenomas to rule out coexistent carcinoma, even if the chance is very remote.

The physiological changes occurring in the breast during pregnancy and lactation make the detection and management of breast abnormalities challenging for clinicians, radiologists and pathologists. Since the first trimester of pregnancy, as lobuloalveolar formation and branching of the lactiferous ducts progress, the glandular/fatty tissue ratio in the breast increases. A thorough breast examination early in pregnancy is essential, since the breasts becomes more firm and nodular in texture during the next months and clinical evaluation more difficult.

Imaging of the pregnant or lactating patient is generally required in the evaluation of a palpable mass, often discovered by the patient herself, or in the presence of a bloody nipple discharge, persistent axillary adenopathy, suspicious abscess or inflammatory disease and pagetoid alterations of the nipple. Routine ultrasound or mammographic screening in asymptomatic pregnant women is not indicated. The first diagnostic tool in every case should be ultrasound, which can easily identify cysts, either simple or complex, galactoceles, lymph nodes and their vascular patterns; these findings usually do not warrant any additional evaluation [5].

In order to limit radiation exposure to the fetus, a mammogram should be performed only in the case of high suspicion of malignancy, to assess for additional lesions or microcalcifications, usually not identified at ultrasound. Even without abdominopelvic shielding, the dose to the fetus from a four-view mammogram has been calculated to be 0·4 mrad, much less than the background radiation dose the fetus receives daily; a dose of 10 rad or greater has been shown to cause fetal malformations.

In pregnant women, mammography demonstrates an overall increase in breast size and parenchymal density with a prominent ductal pattern. Post-lactating patients usually show a return to the original non-pregnant status within 1 to 5 months after stopping lactation; some residual increased density may persist for several months as a result of retained milk products [6]. When indicated, fine needle aspiration or core biopsy should be promptly performed and not delayed until after delivery; the indications are the same for the pregnant or lactating patient as for the non-pregnant and include complex cysts, solid masses, suspicious microcalcifications and persistent inflammatory alterations. Cost-effectiveness of these minimally invasive procedures, compared to more expensive diagnostic tools, should always be in favour of their use. Unfortunately, microbioptic procedures during pregnancy and puerperium often fail to exclude malignancy. A potential complication of breast core biopsy or open surgery that is unique to the lactating patient is milk fistula; in our case, a milky secretion through the surgical wound was drained 10 days after surgery, but ceased after a few days.

Only a few studies have studied the issue of the importance of breast magnetic resonance imaging in pregnant and lactating women [7,8]. Our experience is that MRI may play an important role in the diagnostic evaluation and better definition of a breast solid lesion in the post-partum period and during puerperium, even though the number of cases is still too little to draw any definitive conclusion. The rational behind its use in selected cases during puerperium is based on unsatisfactory reliability of microbioptic procedures [9,10]; another potential indication should be the patient's refusal to undergo diagnostic percutaneous procedures in case of suspicion, as in the case reported here.

A major concern related to the use of MRIs in pregnant patients is safety: fetal exposure to the three main components of the diagnostic procedure (static magnetic fields, pulsed radiofrequency fields and time-varying gradient electromagnetic fields) is still under evaluation. The international standard expresses caution for imaging pregnant women and states that there is no conclusive evidence to establish safety [11]. Until more data are available, the use of MRI during pregnancy should be carefully planned in selected cases only.

An aspect of the present case worthy of mention is that the patient noted the lump during the first trimester of gestation, even though she reported that finding to the physician only during the third trimester, after a progressive increase in the size of the lesion. The evidence so far is that lactating adenomas are typical of the third trimester of pregnancy and no other author in our search reported an earlier onset during gestation; this observation could add elements of uncertainty about the aetiology of lactating adenomas [12].

## Conclusion

The majority of pregnancy-associated breast masses are benign; still, a thorough and prompt evaluation of any lesion during this time is required, in order to rule out a malignancy. Traditionally, in the presence of a solid mass at ultrasound, biopsy has been advised. Lactating adenomas are the most prevalent pregnancy-associated breast lesions; although most lactating adenomas spontaneously involute, the diagnosis is not always straightforward and surgical resection may be required for a definitive diagnosis. In this diagnosis of exclusion, a special role can be attributed to the magnetic resonance image, particularly in the puerperium. Most of all, MRI could help clinicians to avoid unnecessary surgical procedures when ultrasound and microbioptic evaluation have not been conclusive. Further studies and a larger series are needed in order to establish whether MRI can reduce the number of biopsies, both percutaneous and surgical, performed in this subset of patients.

## Abbreviations

US: ultrasound; MRI: magnetic resonance imaging; SPGR: spoiled gradient-recalled; STIR: short tau inversion recovery; MIP: maximum intensity processing; MPR: myocardial perfusion reserve.

## Consent

Written informed consent was obtained from the patient for publication of this case report and accompanying images. A copy of the written consent is available for review by the Editor-in-Chief of this journal.

## Competing interests

The authors declare that they have no competing interests.

## Authors' contributions

MC and PB performed the diagnostic exams and provided the radiological images presented in the paper. The other authors were involved in the preparation of this manuscript. All authors read and approved the final manuscript.
